# Cluster-MLP: An Active Learning Genetic Algorithm Framework for Accelerated Discovery of Global Minimum Configurations of Pure and Alloyed Nanoclusters

**DOI:** 10.1021/acs.jcim.3c01431

**Published:** 2023-10-12

**Authors:** Rajesh K. Raju, Saurabh Sivakumar, Xiaoxiao Wang, Zachary W. Ulissi

**Affiliations:** † Chemical Engineering Department, Carnegie Mellon University, Pittsburgh, Pennsylvania 15217, United States; ‡ School of Chemistry, University of Birmingham, Birmingham B15 2TT, United Kingdom

## Abstract

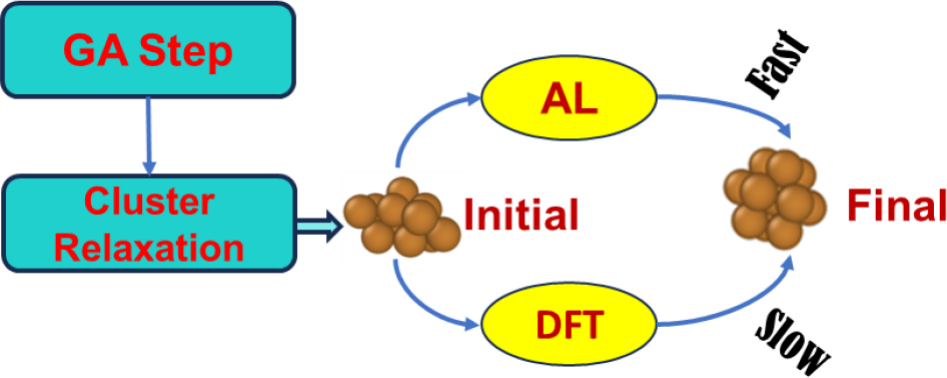

Structural characterization of nanoclusters is one of the major challenges in nanocluster modeling owing to the multitude of possible configurations of arrangement of cluster atoms. The genetic algorithm (GA), a class of evolutionary algorithms based on the principles of natural evolution, is a commonly employed search method for locating the global minimum configuration of nanoclusters. Although a GA search at the DFT level is required for the accurate description of a potential energy surface to arrive at the correct global minimum configuration of nanoclusters, computationally expensive DFT evaluation of the significantly larger number of cluster geometries limits its practicability. Recently, machine learning potentials (MLP) that are learned from DFT calculations gained significant attention as computationally cheap alternative options that provide DFT level accuracy. As the accuracy of the MLP predictions is dependent on the quality and quantity of the training DFT data, active learning (AL) strategies have gained significant momentum to bypass the need of large and representative training data. In this application note, we present Cluster-MLP, an on-the-fly active learning genetic algorithm framework that employs the Flare++ machine learning potential (MLP) for accelerating the GA search for global minima of pure and alloyed nanoclusters. We have used a modified version the Birmingham parallel genetic algorithm (BPGA) for the nanocluster GA search which is then incorporated into distributed evolutionary algorithms in Python (DEAP), an evolutionary computational framework for fast prototyping or technical experiments. We have shown that the incorporation of the AL framework in the BPGA significantly reduced the computationally expensive DFT calculations. Moreover, we have shown that both the AL-GA and DFT-GA predict the same global minima for all the clusters we tested.

## Introduction

Nanocatalysis, more specifically catalysis by nanoclusters, has gained significant momentum as an alternative to the traditional bulk material-based catalysts owing to their high activity, selectivity, and low-metal cost.^1−4^ Recently, nanoalloys (or alloy nanoclusters) have attracted considerable attention in the domain of nanocatalysis.^5,6^ Nanoalloys exhibit different geometrical preferences and physical and chemical properties which in turn impart unique catalytic activity and selectivity compared to pure metal nanoclusters. Tuning the synergistic effect arising from the mixing of multiple metal components is an efficient way for the design of novel nanocluster-based materials by varying composition, size, and atomic ordering.

The major challenge in the design of nanocatalysts is the structural characterization of nanoclusters due to their complex potential energy surface (PES). The number of local minima on the PES rises exponentially with the number of atoms in the nanoclusters often with very similar energies. Moreover, the PES will be even more complex for nanoalloys owing to the multitude of possible arrangements for a given nanoalloy composition. Several algorithms such as the genetic algorithm (GA), basin-hopping (BH), simulated annealing (SA), artificial bee colony (ABC), and particle-swarm method (PS) are commonly employed global optimization (GO) methods for locating the global minimum (GM) of nanoclusters.^7−11^ GA can be considered as a form of basin-hopping with genetic moves, which is a Lamarckian GA as it involves a minimization step of the child geometries after each generation.^12,13^ All these GO methods involve the evaluation of a large number of candidate structures, and the efficacy of these methods is determined by the accuracy and scalability of the methods. Molecular mechanics (MM) methods based on classical interatomic potentials or semiempirical potentials derived from experimental and/or electronic structure methods are widely employed for geometry relaxations due to their low computational cost. Although these methods can be used to predict the GMs of nanoclusters consisting of a few hundred of atoms, they often fail to accurately distinguish between competing geometries with similar energy values due to the large number of approximations incorporated in deriving such empirical potentials and often results in unreliable global minimum (GM) predictions. The density functional theory (DFT) method is an ideal choice for achieving highly accurate geometries and energy values albeit with high computational cost. Evaluations of large number of candidate geometries at the DFT level are a major practical challenge in GO search owing to the greater computational cost associated with the DFT.

To circumvent the large computational cost for the traditional DFT approach, recently, there has been a significant interest in the development of machine learning potentials (MLPs) that are learned from DFT calculations.^14^ For instance, Zhai and co-workers reported a global optimization approach based on the deep neural network (DNN) for modeling metal clusters.^14^ Several MLPs have been developed to date including the most commonly used Behler–Parinello high dimensional neural network (HDNN), Gaussian approximation potential (GAP), spectral neighbor analysis potentials (SNAPS), atomistic machine-learning package (AMP), and moment tensor potentials.^15−19^

Although machine learning potential (MLP) can predict energies and forces much faster than the traditional DFT methods, the accuracy of the MLP predictions is dependent on the quality and quantity of the training data. Active learning (AL) strategies have been implemented to bypass the need of a large and representative training data.^20−22^ AL frameworks allow the MLP to start with little to no training data, and query DFT points when necessary. The MLP can be trained on-the-fly and will be more accurate as more training points are sampled. Active learning approaches have been successfully applied to nanocluster’s GM geometry search.^23−25^ For instance, Wang et al. have demonstrated that on-the-fly machine learning can significantly accelerate the genetic algorithm (GA) search for aluminum nanoclusters employing moment tensor ML potentials.^23^ Hansen and co-workers have developed a symmetry-constraint genetic algorithm coupled with a neural network potential for predicting the energies of Pt–Ni nanoalloys of various sizes, shapes, and compositions with an intend to speed up the calculation.^24^ Except for this work with symmetry-constraint GA, earlier reports on AL approaches in GA methods are applicable only to pure nanoclusters. In our work, we have used Birmingham parallel genetic algorithm (BPGA), a GA approach for locating the GM configurations of both pure and alloyed nanoclusters at the DFT level.^26,27^ Moreover, unlike previous GA methods which are limited to a limited number of mutation operations, the BPGA has many diverse mutation operations that can generate a large number of diverse geometries during the GA search. This is particularly important to avoid stagnation of the GA pool.

## Code Implementation

### Implementation of BPGA into ASE and DEAP Frameworks

In this application note, we introduce Cluster-MLP, our AL approach to the modified version of the BPGA (AL-BPGA) for locating the GM configurations of pure and alloyed nanoclusters.^26,27^ We have used the GIGA version of the BPGA for our work with modifications.^27^ BPGA-GIGA is a parallel genetic algorithm written in Python and utilizes a pool-based methodology for direct DFT global optimization, allowing structural characterization of pure and alloyed nanoclusters. The original BPGA code includes lots of intermediate modules and is not user-friendly for updating or modifying the existing code. In addition, the GIGA version includes separate modules for different electronic structure packages. To make it more user-friendly and easily readable, we first incorporated the BPGA code into the atomistic simulation environment (ASE) interface. ASE provides a more flexible Python framework for setting up, simulating, and analyzing the GA search.^28^ Most importantly, many electronic structure codes or force fields can be easily coupled with the ASE interface to compute various molecular and material quantities such as energy, forces, stresses, etc. Moreover, standard computational tasks such as geometry optimization, molecular dynamics, molecular mechanics, periodic boundary calculations, constrained relaxations, and transition state calculations can be easily performed with various electronic structure software packages through the ASE interface.

The modified BPGA in the ASE format was then incorporated into distributed evolutionary algorithms in Python (DEAP), a novel computational framework for evolutionary algorithms.^29^ The DEAP framework incorporates all the basic data structures and genetic and selection operators as well fitness evaluations and provides a novel user-friendly platform for users to test and implement an evolutionary algorithm quickly or to develop more advanced variants of the genetic programming. In addition, DEAP supports parallelization such as multiprocessing and SCOOP (Scalable COncurrent Operations in Python).

**Figure 1:**
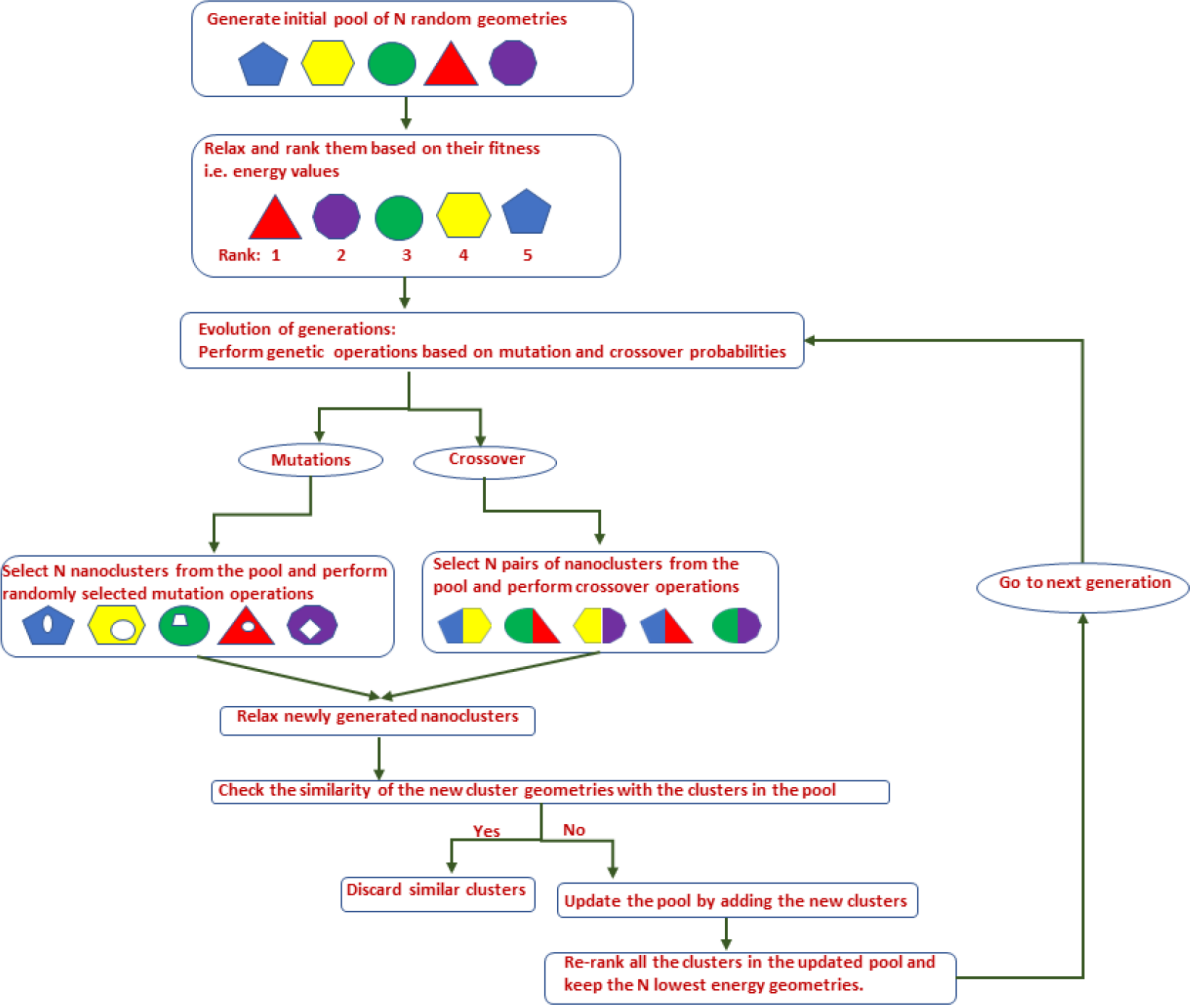
A schematic representation of the DEAP-BPGA algorithm. We have implemented the BPGA with modifications into DEAP, an evolutionary computational framework.

A schematic representation of the modified version of the BPGA is shown in Figure 1. Initially a fixed number of random geometries (10 in this study) were generated and were then relaxed into their local minima forming the initial pool of geometries termed as pool population. These geometries were then ranked based on their fitness values, i.e., energy values. Mutations and crossover operations were then applied to the members of the population forming new diverse geometries. The candidate structures for mutations and crossover operations were selected using a tournament selection method. We used different mutation operations from BPGA such as “move”, “rotate”, “twist”, “partial inversion”, “rattle”, etc., which are registered into the DEAP framework. In the original BPGA version, each generation involves either a mutation or crossover operation. In our Cluster-MLP model, the BPGA code was modified to incorporate multiple mutations or crossover operations in each generation. With this modification, we can perform parallel relaxations of multiple geometries for GA search. Moreover, the user can perform GA searches at both DFT and AL levels (DFT-GA and AL-GA, respectively) in our Cluster-MLP code.

### Active Learning Framework

To accelerate the geometry optimization process in the GA workflow, we utilized a MLP to predict the energy and forces at each single step in the relaxation trajectory, with an AL framework that queries DFT data points and retrains the MLP model during the optimization process. The MLP used in this work was Fast Learning of Atomistic Rare Events Plus Plus (FLARE++), a sparse Gaussian process regression interatomic potential developed by Vandermause et al.^30,31^ Details about the descriptors, kernel functions, and hyperparameters can be found in the original paper. In this work, we used a normalized dot product kernel raised to the second power. The hyperparameters, including the regularization parameter quantifying the variance of the learned energy, and the energy and force noises can be found in Table S1. We found that this set of hyperparameters worked for all testing systems.

**Figure 2:**
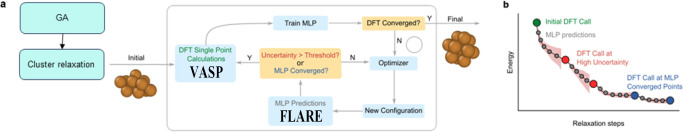
(a) A schematic representation of the active learning (AL) framework. (b) Demonstration of a typical AL geometry relaxation.

The AL framework is illustrated in Figure 2. Each GA step involves the relaxation of a new nanocluster geometry either via a random generation process in the initial pool filling or via a mutation or crossover operation in the existing pool of geometries. For every initial atomic configuration of the cluster, the single point DFT energy and forces are calculated to initialize the MLP. Then, the atomic configuration is updated according to the optimization method, i.e., Broyden–Fletcher–Goldfarb–Shanno (BFGS), and the surrogate model makes force predictions on the new configuration. The FLARE++ potential will provide both the mean value and the uncertainty of the prediction. A DFT single point calculation will be triggered if the relaxation is converged based on MLP forces, or if the uncertainty of the force prediction is higher than the threshold. In this work, the threshold is set as the maximum value of the following: a dynamic tolerance which is 10% of the maximum predicted force and a static tolerance at 0.08 eV/Å. The dynamic tolerance represents the steepness of the surrogate PES, and when it dominates, the model is allowed to be more uncertain as long as it is moving ”downhill”. The static tolerance serves as a preventive measure for the algorithm from calling the DFT too frequently when the predicted forces are small, especially at the end of the relaxation. A typical relaxation process is illustrated in Figure 2b. The majority of the images along the trajectory are evaluated by MLP. Another strategy we implemented for the BFGS optimizer is to reinitialize the Hessian matrix after retraining the MLP. More specifically, after every DFT calculation, the surrogate model is retrained with the new DFT data point, and the model is updated. This updated model makes force predictions on all the previously MLP evaluated images. The entire trajectory is then replayed, and the Hessian matrix is updated with a mixture of the new forces from the surrogate model and the DFT forces. The reinitialized Hessian serves as a better estimation of the local curvature and speeds up the optimization process.

## Results and Discussion

**Figure 3:**
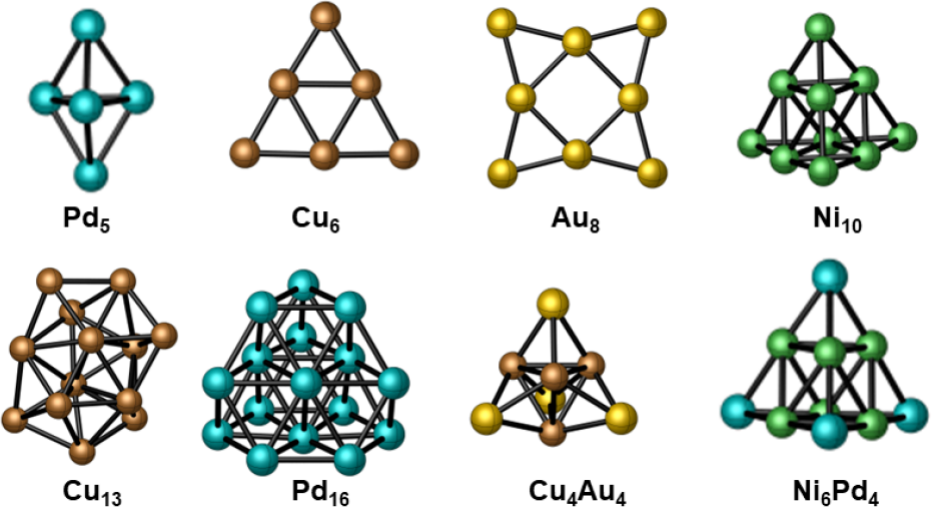
Global minimum configurations of nanoclusters converged to the same minimum in the potential energy surface by both DFT-GA and AL-GA methods.

We tested our active learning genetic algorithm (AL-GA) for locating the global minimum for a number of nanoclusters. We selected a mixture of pure and alloyed nanoclusters, Pd_5_, Cu_6_, Au_8_, Ni_10_, Cu_13_, Pd_16_, Cu_4_A_4_, and Ni_6_Pd_4_, for illustrations. The global minimum configurations for these nanoclusters are shown in Figure 3. We have also confirmed the global minimum configurations for all the nanoclusters employing DFT-GA.

We ran the AL-GA for a total of 10 generations for Pd_5_ and Cu_6_, 15 generations for Au_8_, and 25 generations for Ni_10_, Cu_13_, Pd_16_, Cu_4_A_4_, and Ni_6_Pd_4_. In each generation, 10 new geometries were generated via crossover or mutation operations. After eliminating similar and dissociated nonbonded geometries from these newly generated geometries, the remaining geometries were relaxed with the AL method. We have reaffirmed that all AL relaxed geometries obtained in the AL-GA search were indeed local minimum configurations by performing reoptimizations at the DFT level. GO searches employing both AL-GA and DFT-GA methods result in the same global minimum configurations for all nanoclusters, and it validates the accuracy of our AL-GA method. In addition, we have performed DFT relaxations on the newly generated geometries starting from the initial configurations for a direct comparison with AL-GA and DFT-GA. We have noticed that although both AL and DFT relaxations start from the same initial configurations, most often AL relaxations converge into a different local minimum configuration compared to the local minimum attained through DFT. The difference arises due to the adaption of the different optimization strategies used in DFT and AL relaxations. We used a conjugate gradient optimizer in DFT relaxations while the BFGS algorithm was used in AL relaxations. We used the terminology “similar” and “dissimilar” nanoclusters for differentiating clusters converged to the same and different local minimum configurations by DFT and AL relaxation methods. The total number of nanocluster configurations that converged to the similar and dissimilar relaxed configurations by the DFT and AL methods for various nanoclusters are shown in Figure S1. For instance, out of 63 total relaxations performed for Pd_5_, only 32 relaxations converged to the same local minima by both AL and DFT methods. Similarly for Cu_6_, 48 relaxations were converged to the similar cluster configuration from a total of 81 relaxations. We have also noticed that the number of dissimilar relaxed cluster configurations shows dominance over the similar cluster configuration.

**Figure 4:**
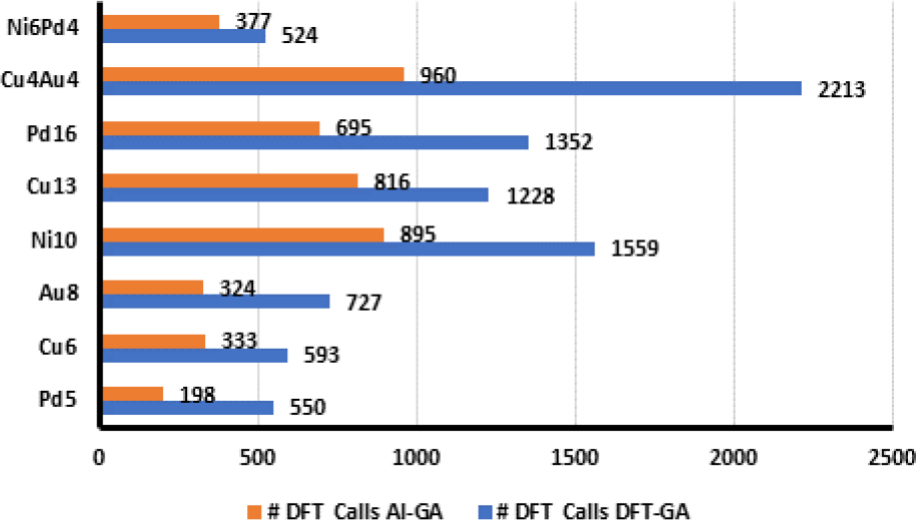
Total number of DFT calls for DFT-GA and AL-GA.

We compared the total number of DFT evaluation steps (or DFT calls) for AL-GA and DFT-GA nanocluster GM searches, as one of the major objectives of implementing AL framework into BPGA is to reduce the number of computationally expensive DFT evaluations in the GA search. We have considered only those relaxations that resulted in the same local minima, i.e., similar clusters for comparing the number of DFT steps in DFT-GA and AL-GA. The total numbers of DFT evaluations performed for AL-GA and DFT-GA relaxations that reached the same local minima are shown in Figure 4.

It is evident from Figure 4 that the incorporation of MLP into the BPGA significantly reduces the number of DFT evaluation steps. For Pd_5_, a total of 550 DFT energy evaluations were performed on 32 cluster relaxations for DFT-GA whereas only 198 DFT energy evaluations were required for AL-GA to arrive at the same local minima from the same initial configurations, a 64% reduction in the total DFT evaluations. Similarly, we observed a reduction of around 43%–72% in DFT evaluation steps for other nanoclusters. For 21 nanocluster relaxations that reached the same local minima for Ni_6_Pd_4_, AL-GA took only 377 DFT evaluations whereas DFT-GA performed a total of 524 DFT steps. Cu_13_ shows a reduction of 66% DFT evaluations for AL-GA for 84 cluster relaxations that reached the same local minima. For the 72 relaxations that reached the same local minima for the nanoalloy cluster Cu_4_Au_4_, the DFT-GA has to perform a total of 2213 DFT steps. On the contrary, the AL-GA relaxations required only 960 DFT evaluations for these 72 relaxations, a reduction of 43% in the total DFT steps.

We have also made structural analyses on the relaxed geometries predicted by the AL and DFT methods. As discussed above, we have only considered the relaxed geometries converged to the same local minima, i.e., similar clusters for comparing the accuracy of the AL relaxations with respect to the DFT relaxations. We computed the average absolute difference in the bond distances for AL and DFT relaxed geometries by taking into account all the possible bond distances in each nanocluster. We then calculated the largest deviations as well as the mean absolute deviation (MAD) and root-mean-square deviation (RMSD) for all different nanoclusters, and the values are given in Table S2. It is evident from Table S2 that AL and DFT relaxations converge the geometries to the same minimum in the PES as the MAD and RMSD values for the difference in average bond distances are negligibly smaller values ranging from 0.0038 to 0.0088 for MAD and 0.0052 to 0.0099 for RMSD. Such negligible changes in the average bond distances for AL and DFT relaxed nanoclusters clearly demonstrate the accuracy of our AL method with respect to the high-level DFT method.

Similarly, we have also computed the largest deviations as well as the mean absolute deviation (MAD) and root-mean-square deviation (RMSD) for the difference in the energy predicted by the AL and DFT methods and are given in Table S3. The MAD values range from 0.0009 to 0.0025 eV while the RMSD values range from 0.0018 to 0.0035 eV. In addition, the largest deviations in energy values are insignificantly smaller. Such negligible changes in the DFT relaxed geometries and the AL relaxed geometries imply that both GA search methods converge to the same local minima and establishes the accuracy of our active learning approach.

**Figure 5:**
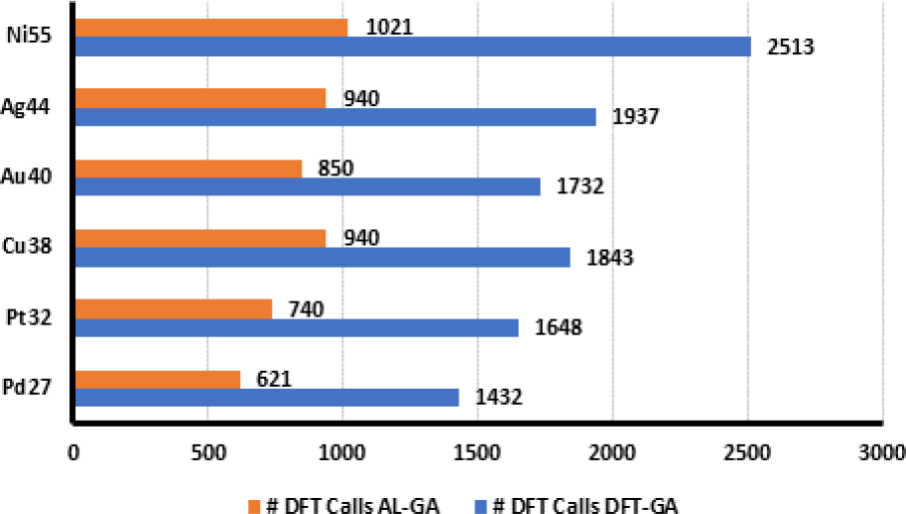
Total number of DFT calls for DFT-GA and AL-GA for 20 cluster relaxations for larger nanoclusters.

The global minimum search for nanoclusters employing DFT-GA is practically limited to clusters with smaller number of atomic compositions owing to the high computational cost associated with the relaxation of larger nanoclusters at the DFT level. To evaluate the efficacy of the AL-GA approach, we have compared the total number of DFT calls required for the relaxation of randomly selected 20 nanoclusters that are converged to the same local minima by both AL and DFT methods for larger nanoclusters (Pd_27_, Pt_32_, Cu_38_, Au_40_ Ag_44_, and Ni_55_), and the results are summarized in Figure 5. For large nanoclusters, we have found similar reductions in the total number of DFT calls for various larger nanoclusters in the range of approximately 49%–60%. For instance, Ni_55_ AL relaxations reduced the total number of DFT calls from 2513 to 1021 for 20 cluster relaxations, approximately 60% reduction in the total DFT evaluation steps. We have found in general that AL-GA framework achieves a reduction of approximately 50%–55% in most of our test cases.

## Conclusions

In short, we have presented Cluster-MLP, a novel active learning GA framework for the faster discovery of global minima of pure and alloyed nanoclusters (multimetallic) employing FLARE++ potential. Cluster-MLP is based on the modified version of the BPGA, and in the current version, we have incorporated ASE and DEAP frameworks into the code which make it more user-friendly. The user can easily modify the existing GA framework for testing their novel ideas without much coding exercise. For instance, the user can add a new mutation or crossover operations or modify the existing operations by simply registering their operations in the DEAP framework. In addition to AL-GA search, the code allows the user to perform DFT-GA searches. We have demonstrated that the inclusion of FLARE++ ML potential into the BPGA has reduced the computationally expensive DFT calls for the GA search for finding the global minimum configurations for pure and alloyed nanoclusters. Moreover, we have established that there are no significant changes in the energies or geometries for AL and DFT relaxed nanoclusters. Our AL-GA framework does not require computationally prohibitive pretraining for potentials, and the user just has to provide the element type and compositions for running the code.

Although the original BPGA is capable of performing global optimization of nanoclusters and nanoalloys on surface supports, presently our AL-GA implementation works only for bare gas-phase nanoclusters and nanoalloys. As a potential future avenue for improvement, we may incorporate BPGA into our AL framework for the global minimum search on nanoclusters and nanoalloys on external supports. Moreover, we may incorporate the AL framework into the GIGA version of BPGA which is capable of performing a GA search on bare or supported nanoclusters and nanoalloys with one or multiple ligands of the same or different types.

## Data Availability

**Code and Software Availabilty.** The code used in this work is available at https://github.com/ulissigroup/cluster_mlp. The software is open-sourced and licensed under a GNU General Public License.

## Supporting Information Available

FLARE potential hyperparameters and results for AL-GA and DFT-GA GM searches (PDF)

## References

[ref1] Olveira S.; Forster S. P.; Seeger S. Nanocatalysis: Academic discipline and industrial realities. Journal of Nanotechnology 2014, 2014, 1–19. 10.1155/2014/324089.

[ref2] Dai Y.; Wang Y.; Liu B.; Yang Y. Metallic nanocatalysis: An accelerating seamless integration with nanotechnology. Small 2015, 11, 268–289. 10.1002/smll.201400847. 25363149

[ref3] Piccolo L. Restructuring effects of the chemical environment in metal nanocatalysis and single-atom catalysis. Catal. Today 2021, 373, 80–97. 10.1016/j.cattod.2020.03.052.

[ref4] Zhai H.; Alexandrova A. N. Fluxionality of Catalytic Clusters: When It Matters and How to Address It. ACS Catal. 2017, 7, 1905–1911. 10.1021/acscatal.6b03243.

[ref5] Ferrando R.; Jellinek J.; Johnston R. L. Nanoalloys: From theory to applications of alloy clusters and nanoparticles. Chem. Rev. 2008, 108, 845–910. 10.1021/cr040090g. 18335972

[ref6] Jellinek J. Nanoalloys: Tuning properties and characteristics through size and composition. Faraday Discuss. 2008, 138, 11–35. 10.1039/b800086g. 18447006

[ref7] Johnston R. L. Evolving better nanoparticles: Genetic algorithms for optimizing cluster geometries. J. Chem. Soc., Dalton Trans. 2003, 3, 4193–4207. 10.1039/b305686d.

[ref8] Wales D. J.; Doye J. P. Global optimization by basin-hopping and the lowest energy structures of Lennard-Jones clusters containing up to 110 atoms. J. Phys. Chem. A 1997, 101, 5111 10.1021/jp970984n.

[ref9] Hohl D.; Jones R. O.; Car R.; Parrinello M. Structure of sulfur clusters using simulated annealing: S2 to S13. J. Chem. Phys. 1988, 89, 6823 10.1063/1.455356.

[ref10] Zhang J.; Dolg M. ABCluster: The artificial bee colony algorithm for cluster global optimization. Phys. Chem. Chem. Phys. 2015, 17, 24173 10.1039/C5CP04060D. 26327507

[ref11] Lv J.; Wang Y.; Zhu L.; Ma Y. Particle-swarm structure prediction on clusters. J. Chem. Phys. 2012, 137, 084104 10.1063/1.4746757. 22938215

[ref12] Turner G. W.; Tedesco E.; Harris K. D.; Johnston R. L.; Kariuki B. M. Implementation of Lamarckian concepts in a Genetic Algorithm for structure solution from powder diffraction data. Chem. Phys. Lett. 2000, 321, 183–190. 10.1016/S0009-2614(00)00318-3.

[ref13] Bauer M. N.; Probert M. I. J.; Panosetti C. Systematic Comparison of Genetic Algorithm and Basin Hopping Approaches to the Global Optimization of Si(111) Surface Reconstructions. J. Phys. Chem. A 2022, 126, 3043–3056. 10.1021/acs.jpca.2c00647. 35522778 PMC9126620

[ref14] Zhai H.; Alexandrova A. N. Ensemble-Average Representation of Pt Clusters in Conditions of Catalysis Accessed through GPU Accelerated Deep Neural Network Fitting Global Optimization. J. Chem. Theory Comput. 2016, 12, 6213–6226. 10.1021/acs.jctc.6b00994. 27951667

[ref15] Behler J.; Parrinello M. Generalized Neural-Network Representation of High-Dimensional Potential-Energy Surfaces. Phys. Rev. Lett. 2007, 98, 146401 10.1103/PhysRevLett.98.146401. 17501293

[ref16] Behler J. Four Generations of High-Dimensional Neural Network Potentials. Chem. Rev. 2021, 121, 10037–10072. 10.1021/acs.chemrev.0c00868. 33779150

[ref17] Bartók A. P.; Payne M. C.; Kondor R.; Csányi G. Gaussian Approximation Potentials: The Accuracy of Quantum Mechanics, without the Electrons. Phys. Rev. Lett. 2010, 104, 136403 10.1103/PhysRevLett.104.136403. 20481899

[ref18] Thompson A.; Swiler L.; Trott C.; Foiles S.; Tucker G. Spectral neighbor analysis method for automated generation of quantum-accurate interatomic potentials. J. Comput. Phys. 2015, 285, 316–330. 10.1016/j.jcp.2014.12.018.

[ref19] Khorshidi A.; Peterson A. A. Amp: A modular approach to machine learning in atomistic simulations. Comput. Phys. Commun. 2016, 207, 310–324. 10.1016/j.cpc.2016.05.010.

[ref20] Musielewicz J.; Wang X.; Tian T.; Ulissi Z. FINETUNA: fine-tuning accelerated molecular simulations. Machine Learning: Science and Technology 2022, 3, 03LT01 10.1088/2632-2153/ac8fe0.

[ref21] Shuaibi M.; Sivakumar S.; Chen R. Q.; Ulissi Z. W. Enabling robust offline active learning for machine learning potentials using simple physics-based priors. Machine Learning: Science and Technology 2021, 2, 025007 10.1088/2632-2153/abcc44.

[ref22] Yang Y.; Jiménez-Negrón O. A.; Kitchin J. R. Machine-learning accelerated geometry optimization in molecular simulation. J. Chem. Phys. 2021, 154, 234704 10.1063/5.0049665. 34241251

[ref23] Wang Y.; Liu S.; Lile P.; Norwood S.; Hernandez A.; Manna S.; Mueller T. Accelerated prediction of atomically precise cluster structures using on-the-fly machine learning. npj Computational Materials 2022, 8, 64–66. 10.1038/s41524-022-00856-x.

[ref24] Han S.; Barcaro G.; Fortunelli A.; Lysgaard S.; Vegge T.; Hansen H. A. Unfolding the structural stability of nanoalloys via symmetry-constrained genetic algorithm and neural network potential. npj Comput. Mater. 2022, 8, 121 10.1038/s41524-022-00807-6.

[ref25] Bisbo M. K.; Hammer B. Global optimization of atomic structure enhanced by machine learning. Phys. Rev. B 2022, 105, 245404 10.1103/PhysRevB.105.245404.

[ref26] Davis J. B.; Shayeghi A.; Horswell S. L.; Johnston R. L. The Birmingham parallel genetic algorithm and its application to the direct DFT global optimization of IrN (N = 10–20) clusters. Nanoscale 2015, 7, 14032–14038. 10.1039/C5NR03774C. 26239404

[ref27] Jäger M.; Schäfer R.; Johnston R. L. GIGA: A versatile genetic algorithm for free and supported clusters and nanoparticles in the presence of ligands. Nanoscale 2019, 11, 9042–9052. 10.1039/C9NR02031D. 31025685

[ref28] Hjorth Larsen A.; Jørgen Mortensen J.; Blomqvist J.; Castelli I. E; Christensen R.; Dułak M.; Friis J.; Groves M. N; Hammer B.ør.; Hargus C.; Hermes E. D; Jennings P. C; Bjerre Jensen P.; Kermode J.; Kitchin J. R; Leonhard Kolsbjerg E.; Kubal J.; Kaasbjerg K.; Lysgaard S.; Bergmann Maronsson J.; Maxson T.; Olsen T.; Pastewka L.; Peterson A.; Rostgaard C.; Schiøtz J.; Schutt O.; Strange M.; Thygesen K. S; Vegge T.; Vilhelmsen L.; Walter M.; Zeng Z.; Jacobsen K. W The atomic simulation environment—a Python library for working with atoms. J. Phys.: Condens. Matter 2017, 29, 273002 10.1088/1361-648X/aa680e. 28323250

[ref29] Fortin F.-A.; De Rainville F.-M.; Gardner M.-A.; Parizeau M.; Gagné C. DEAP: Evolutionary Algorithms Made Easy. Journal of Machine Learning Research 2012, 13, 2171–2175.

[ref30] Vandermause J.; Torrisi S. B.; Batzner S.; Xie Y.; Sun L.; Kolpak A. M.; Kozinsky B. On-the-fly active learning of interpretable Bayesian force fields for atomistic rare events. npj Computational Materials 2020, 6, 1–11. 10.1038/s41524-020-0283-z.

[ref31] Vandermause J.; Xie Y.; Lim J. S.; Owen C. J.; Kozinsky B. Active learning of reactive Bayesian force fields applied to heterogeneous catalysis dynamics of H/Pt. Nat. Commun. 2022, 13, 5183 10.1038/s41467-022-32294-0. 36055982 PMC9440250

