# Supplementary material for: Cluster-MLP: An Active Learning Genetic Algorithm Framework for Accelerated Discovery of Global Minimum Configurations of Pure and Alloyed Nanoclusters
Source: J Chem Inf Model. 2023 Oct 12;63(20):6192–7. doi: 10.1021/acs.jcim.3c01431 (PMC10598790; doi:10.1021/acs.jcim.3c01431)
Supplement: Supplementary file 1 — ci3c01431_si_001.pdf [file ci3c01431_si_001.pdf]

Supporting Information

Cluster-MLP: An Active Learning Genetic Algorithm Framework for Accelerated Discovery of Global minimum Configurations of Pure and Alloyed Nanoclusters

Rajesh K. Raju,[1,2,*)] Saurabh Sivakumar,[1,†)] Xiaoxiao Wang,[1,†)] and Zachary W. Ulissi[1,*)]

[1)]*Chemical Engineering Department, Carnegie Mellon University, Pittsburgh, PA 15217, USA*

[2)]*School of Chemistry, University of Birmingham, Birmingham B15 2TT, U.K*

[‡)]Electronic mail: zulissi@andrew.cmu.edu; rajeshra@andrew.cmu.edu

[*)]Corresponding author

[†)]Equal contributions

S1. FLARE POTENTIAL HYPERPARAMETERS

TABLE S1. Summary of the hyperparameters for Flare.

Hyperparameter	Description	Value
N_{rad}	The number of radial functions	12
l_{max}	The maximum degree in the spherical harmonics expansion	3
r_{cut}	The cutoff radius	5 Å
σ	The variance of the learned energy	4.5 eV
σ_E	The energy noise	9 meV
σ_F	The force noise	5 meV/Å

S2. RESULTS FOR AL-GA AND DFT-GA GM SEARCHES

TABLE S2. Largest deviation as well as mean absolute deviation (MAD) and root-mean square deviation (RMSD) for average difference in bond distance (Å) for AL and DFT relaxed geometries for different nanoclusters in the AL-GA and DFT-GA GM searches. For each nanocluster, we have averaged the absolute bond distance difference for all the possible bond distances.

Nanocluster	Largest Deviation(Å)	MAD (Å)	RMSD (Å)
Pd_5	0.0154	0.0038	0.0052
Cu_6	0.0142	0.0056	0.0068
Au_8	0.0189	0.0088	0.0099
Ni_{10}	0.0155	0.0057	0.0064
Cu_{13}	0.0200	0.0071	0.0082
Pd_{16}	0.0197	0.0084	0.0094
Cu_4A_4	0.0188	0.0079	0.0089
Ni_6Pd_4	0.0192	0.0072	0.0084

TABLE S3. Largest deviation as well as mean absolute deviation (MAD) and root-mean square deviation (RMSD) for difference in energy values (eV) for AL and DFT relaxed geometries for different nanoclusters in the AL-GA and DFT-GA GM searches.

Nanocluster	Largest Deviation (eV)	MAD (eV)	RMSD (eV)
Pd_5	0.0083	0.0009	0.0018
Cu_6	0.0100	0.0016	0.0029
Au_8	0.0108	0.0015	0.0026
Ni_{10}	0.0185	0.0017	0.0029
Cu_{13}	0.0092	0.0015	0.0024
Pd_{16}	0.0103	0.0025	0.0035
Cu_4A_4	0.0060	0.0014	0.0021
Ni_6Pd_4	0.0068	0.0011	0.0017

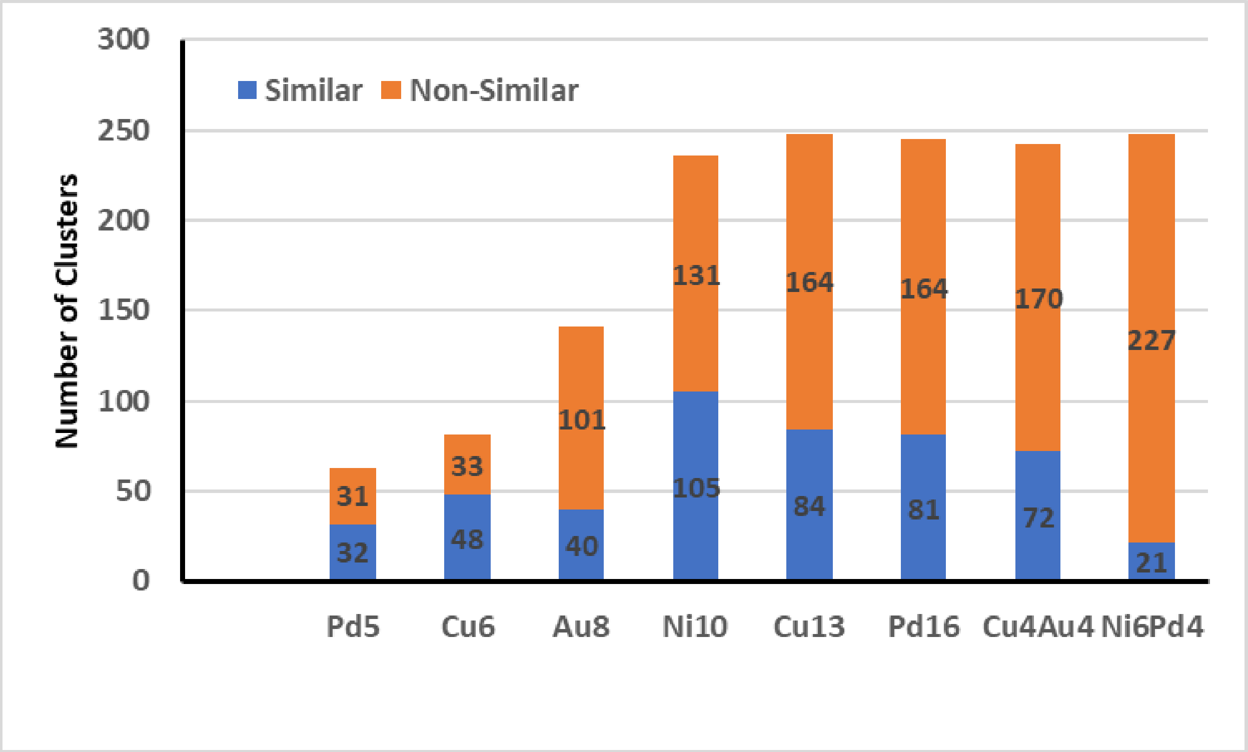

FIG. S1. The number of nanoclusters converged to the same local minima (similar clusters) and different local minima (dissimilar clusters) by the DFT and AL relaxation methods.
